# Development of smooth pursuit eye movements in very preterm infants: 1. General aspects

**DOI:** 10.1111/j.1651-2227.2011.02218.x

**Published:** 2011-07

**Authors:** Katarina Strand-Brodd, Uwe Ewald, Helena Grönqvist, Gerd Holmström, Bo Strömberg, Erik Grönqvist, Claes von Hofsten, Kerstin Rosander

**Affiliations:** 1 Department of Women's and Children's Health, Uppsala universityUppsala, Sweden; 2 Department of Psychology, Uppsala UniversityUppsala, Sweden; 3 Department of Neuroscience, Uppsala UniversityUppsala, Sweden; 4 Institute for Labour Market Policy Evaluation (IFAU) and Department of Economics, Uppsala UniversityUppsala, Sweden

**Keywords:** Infant development, Oculo-motor, Preterm infant, Smooth pursuit eye movements

## Abstract

**Aim:**

To investigate early oculo-motor development in a population-based cohort of very preterm infants.

**Methods:**

Early oculo-motor development was prospectively studied by measuring smooth pursuit eye movements at 2 and 4 months corrected age in a population of very preterm infants born in Uppsala County 2004–2007. Eighty-one preterm infants were studied, and 32 healthy term infants constituted the control group.

**Results:**

The study group consisted of infants with a mean gestational age of 28 + 5 weeks. At 2 and 4 months corrected age, infants born very preterm showed lower gain (p < 0.001) and proportion of smooth pursuit eye movements (p < 0.001) compared to the control group. The boys showed higher gain of smooth pursuit eye movements at both 2 and 4 months corrected age, compared to girls.

**Conclusions:**

Oculo-motor development measured by smooth pursuit eye movements is delayed in very preterm infants at 2 and 4 months corrected age. This might be a risk factor or early indicator of later perceptual and behavioural impairment.

## Introduction

During the recent decades, the incidence of infants born very preterm [i.e. born before 32 gestational weeks (GW)] has increased, and today, infants born before 27 GW have a survival rate that is approaching 70–80% (1).

Although there is a significant risk of developing major handicaps such as cerebral palsy, hearing loss, blindness and/or mental retardation among the survivors (2,3), the prevalence of these handicaps has not increased with the increasing survival rate. However, later disabilities, such as perceptual, coordinative and cognitive impairment, have been reported in 15–45% of very preterm infants, which raises concern (4,5). The differences in reported incidences may reflect differences in population characteristics, methods used for assessment and medical treatment, ages at which the infants were examined and/or the socioeconomic status of the mothers/parents.

Perceptual, coordinative and cognitive impairments are often subtle and difficult to diagnose during the infant's first years of life, but may emerge as increasing problems with coordination, social interaction, focused attention and learning at school age (6). High quality of vision is an essential prerequisite to perform activities in daily life (7,8). Delayed or impaired development of visual perceptual capacity might therefore be an important confounder.

Visual development progresses rapidly immediately after birth and continues to be fast during the infant's first year of life, when brain development is intense (9) and the assessment of visual capacity early in infancy could therefore be a sensitive measure of delayed development. In particular, the capability to detect motion direction and to smoothly track moving objects is considered as an important part of the attention mechanisms. Smooth eye-tracking movements are essential for focusing gaze on moving objects. In the newborn infant, eye tracking is mostly saccadic but at 6–8 weeks of age, the capacity to track objects begins to develop and smooth pursuit reach a level almost equal to that of an adult at about 4–5 months of age in term infants (10). The development results in precise smooth pursuit that predictively stays on the moving object.

**Key notes**

- Development of smooth pursuit eye movements at 2 and 4 months corrected age was delayed in a population of very preterm infants compared to a control group of full term infants.

**Figure 1:**
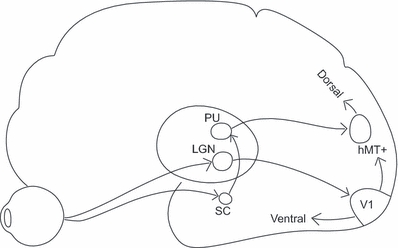
Visual pathways in the brain. LGN = lateral geniculate nucleus; PU = pulvinar nucleus; SC = superior colliculus; DORSAL = the dorsal pathway; VENTRAL = the ventral pathway = hMT+ = the human medial temporal complex area; V1 = primary visual cortex.

There are two main pathways that process visual motion information: the primary pathway and the subcortical stream (Fig. 1). In the primary pathway, the magnocellular cells process information of motion of subjects (‘where’) from the optic nerve via the lateral geniculate nucleus and the primary visual cortex (V1) in the occipital lobe area to associative visual cortex in the parietal lobe, i.e. the dorsal stream. [In the ventral stream, information of ‘what’ is processed to associative cortical areas in the temporal lobe (11,12).] In addition to this thalamo-cortical pathway, motion is also processed in the subcortical stream that goes from the retina via superior colliculus (SC) and pulvinar nucleus (PU) to the human medial temporal complex area (hMT+), bypassing V1. This subcortical stream has been found to function early in life (starting before 2 months of age). For development of smooth pursuit, it is necessary that the functioning of the human medial temporal complex area in the cerebral cortex has developed. In this area, motion direction is specified and further neural processing takes place via a broad neural network, and thus, the human medial temporal complex area is considered as the gateway to the dorsal stream and is essential for smooth pursuit (13).

The complexity in this network suggests that even small injuries, growth delay, and indirect effects of inflammation or vascular damage may have substantial detrimental effects. It has been shown that periventricular leukomalacia (PVL) resulting from hypoxic injuries in the periventricular white matter in preterm infants often induces impact on this network area (14). In addition, visual information of motion is transferred to motor areas, and this sensorimotor coupling is another vulnerable part of the developing brain.

We hypothesize that the development of smooth pursuit is delayed or impaired in very preterm infants. Furthermore, we speculate that such delay may predict developmental problems in the very preterm infant. For this purpose, the ‘LOVIS project’ was initiated to follow a population of very preterm-born infants until adult age regarding visual perception, ophthalmological parameters and neuromotor, cognitive and psychosocial development.

## Aim

The aim of the present study was to evaluate early visuomotor development in a population-based cohort of infants born before 32 GW. Infants’ visuomotor capacity regarding smooth pursuit at 2 and 4 months corrected age (CA) was measured and compared to healthy term infants at the same ages. In addition, background clinical data including major neonatal complications are recorded for the cohort.

## Methods

The LOVIS project is a multidisciplinary population-based follow-up study involving the Departments of Women's and Children's Health, Psychology and Neuroscience at Uppsala University.

### Subjects

In Uppsala County (population 320 000), all deliveries take place at Uppsala University Hospital. During a 4-year study period 2004–2007, 145 of a total of 14 532 live-born infants were born at 22+0–31+6 GW. After primary treatment in the delivery room by a neonatal team, all live-born infants were admitted to and cared for in the Neonatal Intensive Care Unit, Uppsala University Children's Hospital.

The infants were recruited into the study after the most intensive initial period, usually at around 2 weeks postnatal age. Parental written consent was obtained after written and oral information. Ethical approval for this study was received from the human research Ethical Committee of the Medical Faculty at Uppsala University (nr Ups 03-665). Sixteen infants died before recruitment, three infants moved, nine did not participate because of parental refusal and two infants were missed. Two infants with syndromes including mental retardation, one with Down syndrome and one with Bartters’ syndrome, were excluded. Thus, 113 very preterm infants (=89% of the surviving infants without syndromes) constituted the study population. Two of the infants died after recruitment but before discharge; one because of severe primary pulmonary hypertension and the other after extensive intestinal surgery, leaving a cohort of 111 infants for follow-up (flowchart). Of these, 101 infants were called to Babylab at the Department of Psychology, Uppsala University. Thirteen parents declined to participate because of logistical problems and seven infants were too fussy during the investigation, leaving a group of 81 very preterm infants to be measured. The infants in the control group were recruited via public birth records and the families were contacted by mail, according to the routines at Babylab. Thirty-five healthy infants born at term were investigated, three were excluded because of inattention and technical problems leaving a group of 32 healthy term–born infants, 17 boys and 15 girls.

**Figure:**
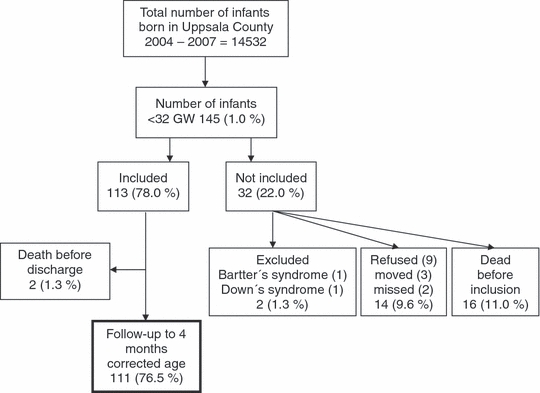
Flow chart over study population.

Gestational length was estimated by ultrasound at gestational week 16–18.

The study group (n = 113) had a mean gestational age of 28+5 GW (range 22+0–31+6), 35 (31%) of the infants were born before 28+0 GW. Mean birth weight was 1198 g (range 520–2030). The mean age of the 95 mothers was 31.8 years (range 18.3–46.5) and 65 (61%) were primipara. Of the 113 infants, 35 (31%) were twins or triplets. Sixty-one (54%) of the infants were boys and 52 (46%) were girls.

Prenatal steroid treatment for lung maturation was given to 78 (82%) of the mothers. Premature rupture of membranes (PROM) >24 h occurred in 15 (16%) of the pregnancies. Sixty-eight (60%) of the infants were born by caesarean section. Twenty-three (20%) of the infants were born small for gestational age (SGA), i.e. infants with a birth weight of more than two standard deviations (SD) below the mean in accordance with the national foetal weight-based growth standard (15).

### Neonatal complications

Bronchopulmonary dysplasia (BPD) was defined as a need for >25% oxygen treatment to achieve saturation >90% at 36 weeks postmenstrual age (16).

Necrotizing enterocolitis (NEC) was defined according to criteria defined by Bell et al. (17).

Retinopathy of prematurity (ROP) was defined according to the International Classification of ROP (18). Screening for ROP was performed by paediatric ophthalmologists weekly from 5 weeks postnatal age until the retina was fully vascularized, i.e. around term age or until regression of ROP. Treatment indications were in accordance with recommendations of the ETROP study (19).

Ultrasound of the brain for detection of intraventricular haemorrhage (IVH) and PVL was performed by paediatric radiologists using an Acuson Sequoia 512 (Siemens Medical™, Malvern, PA, USA) and a 10 Hz probe, at 2–7 days postnatally and at 35 weeks postmenstrual age. IVH was defined according to Papile (20). PVL was defined in size (mm), laterality and as cystic or diffuse (21,22).

### Smooth pursuit eye movements

Smooth pursuit eye movements were evaluated as proportion of smooth pursuit (propSP) and gain of smooth pursuit (Gain)*.* PropSP measures the proportion of the total eye movement that consists of smooth pursuit, and Gain measures the proportion of the object's movement that is followed by smooth pursuit.

#### Apparatus

**Figure 2:**
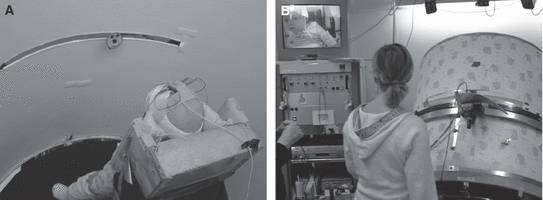
The apparatus for measuring eye movements from interior of the cylinder (A) and exterior (B).

The measurements were performed in a specially designed apparatus described earlier (10). Briefly, it consisted of a cylinder of 1 m diameter and height and with an opening sector. In the centre of the cylinder, an infant chair was placed, inclined at 40°. The cylinder created a visual surrounding that prevented distractions and allowed the subject to concentrate during the trials. In the cylinder wall, a narrow horizontal slit faced the infant. In the slit, an object (a bright orange coloured happy face, 7 cm diameter) with a small video camera in its centre could be moved according to a certain motion pattern and amplitude (Fig. 2A, B). When the object moved, it was always 50 cm from the centre of the cylinder. The video camera gave the parent constant information on their infant during the trials. The recordings were used to check that the infant was attentively tracking the object's motion. In the present study, two motion functions (sinusoidal and triangular with constant velocity) and two amplitudes (10 or 20°) were used. The velocity was 0.25 Hz, corresponding to a constant speed of 12.5 and 25°/s in the case of the 10° and 20° amplitude, respectively. In the case of the sinusoidal motion, the maximum speed was 20 and 40°/s for the 10° and 20° amplitude, respectively.

#### Measurements

An opto-electronic motion recording system (Qualisys, Proreflex, Gothenburg, Sweden) was used to measure the object motion and the motion of the infant's head. Passive reflective markers (5 mm in diameter) gave reflections in the infrared range to light-sensitive cameras that were placed in the ceiling above the infant. One marker was placed on the moving object and three on the surface of the skull (one midsagittal, two coronal). Measurement frequency was set to 240 Hz and was fed into custom-made software for later analysis. The horizontal eye movements were measured in synchrony with these measurements. Electro-oculographic (EOG) recordings were performed using an amplifier system designed by G.Westling (Department of Physiology, Umeå University, Umeå, Sweden). Miniature skin electrodes (Beckman) were attached to the outer canthi and the ground to the ear lobe or the forehead. Every sequence of measurement trial was set to 35 s.

#### Procedure

When the parent(s) arrived at the laboratory, they were informed of the procedure and signed a written consent form according to the Helsinki declaration 1964. The infant was placed in the seat in the cylinder with the EOG electrodes and the reflective markers properly attached. After positioning the cylinder with the slit in front of the infant, the first trial (a calibration of the EOG) started immediately, followed by four trials (two amplitudes and two motion patterns). If the infant was distressed or fussed, the trial was immediately interrupted and the parent was able to comfort their infant before the experiment continued. Overall, the infants tolerated the procedure very well and were attentively looking during the trials.

The video tapes were examined to decide whether a trial could be further analysed. In the Qualisys software, the positions of the markers were transformed to coordinates in space (x, y, z and time) and the EOG signals to coordinate in the plane perpendicular to the cylinder axis (x and time). All data were fed into a calculation program in FYSTAT (Department of Physiology, Umeå University, Umeå, Sweden) or Matlab (Mathworks Inc™, Natick, MA, USA).

#### Data analysis

**Figure 3:**
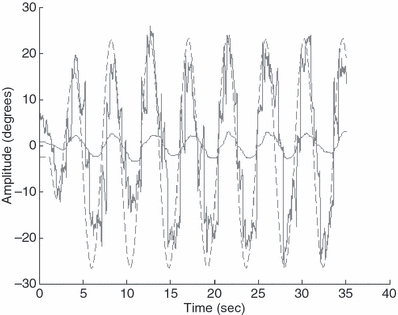
Examples of registrations of eye and head movements and object motion (dashed) in an infant at 9 week corrected age. The infant was born at 28 week GA.

The gaze was estimated as the sum of eye and head position. Angular velocities were estimated as the difference between consecutive coordinates. Each velocity value was the mean difference between four time samples corresponding to a 50-Hz filter. The velocities of the eyes, the head and the target were routinely calculated in this way. Saccades were defined as periods of the recorded eye velocity higher than 50°/s of the tracking velocity amplitude. To calculate the smooth pursuit component, the saccades were eliminated from the composite, raw eye movement record. The periods cut out were replaced with interpolated data (10). Average amplitude for smooth pursuit was calculated. The ratio between smooth pursuit and object amplitude was calculated (Gain) and further the ratio of amplitude of smooth pursuit to that of the raw eye movements was estimated (propSP). In Fig. 3, an example of eye–head tracking of a VPT infant (born at 28 gw) is shown. It can be observed that the infant tracks the object mainly with saccades at this age and that small head movements accompany the eye movements.

### Statistics

Statistical analyses were performed using spss 16.0 (SPSS Inc, IBM, Chicago, IL, USA) and Stata/SE 11.0 (StataCorp LP, College Station, TX, USA). The mean proportion and gain at 2 and 4 months CA was calculated and independent *t*-test was used to test differences between groups. To further investigate differences between the study and full-term groups, a linear regression according to the following model was estimated: 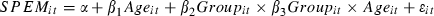


The outcome smooth pursuit is measured during the experiment for infant *i* at time *t*. The model examines the relationship between an indicator for the two (corrected) age groups (Age = 0 (1) at 2 (4) months), between an indicator for very preterm and full term (Group = 0 (1) for full term/very preterm) and an interaction effects between *Group* and *Age.*

The distribution of data was displayed using an estimated kernel density (23), where the contribution of each data point is smoothed out nonparametrically over a local neighbourhood around that data point to create a density curve. To test whether the share of PT infants reaching different parts of the distribution for full-term infants changed between 2 and 4 months of (corrected) age, an indicator of whether the PT infant reached the 10th or the 50th percentile of the full-term group is regressed.

## Results

### Neonatal complications

Bronchopulmonary dysplasia occurred in 23 infants (20%), IVH in 24 (21%) infants, IVH >grade 2 in four infants (3.5%) and PVL in seven infants (6%) with cystic PVL in three infants (2.5%). ROP occured in 32 infants (28%) and ROP >grade 2 in nine infants (8%). None of the infants were diagnosed with NEC. In 84 (74.4%) of all 113 LOVIS infants, no major neonatal complications, i.e. BPD, IVH >grade 2, PVL, NEC and/or ROP >grade 2 were found.

### Smooth pursuit eye movements

Smooth pursuit eye movements were investigated in 81 very preterm infants and 32 full-term infants and were evaluated as proportion of smooth pursuit to raw eye movement (propSP) and gain of smooth pursuit as a ratio of the object movement (Gain).

**Figure 4:**
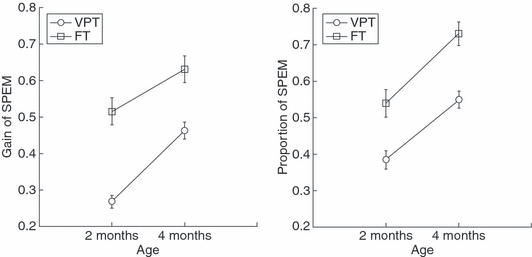
Proportion and gain of smooth pursuit in full-term (squares) and very preterm (circles) infants at 2 and 4 months of corrected age.

There was no significant difference between the very preterm infants participating vs. those not participating in terms of gestational age, birth weight, gender or neonatal complications. Measurement at both 2 and 4 months was performed in 48 of the very preterm and 20 of the full-term infants. Measurement at 2 months only was performed in 10 very preterm and nine full-term infants, and measurement at 4 months only was performed in 23 very preterm and three full-term infants. At 2 months CA, mean propSP and mean Gain were higher in the full-term group compared to the very preterm group (0.54 vs. 0.37, p = 0.001, η^2^ = 0, 13 and 0.52 vs. 0.27, p < 0.001, η^2^ = 0.36, respectively). At 4 months CA, mean propSP and mean Gain were also higher in the full-term group when compared to the very preterm group (0.73 vs. 0.55, p < 0.001, η^2^ = 0, 15 and 0.63 vs. 0.46, p = 0.001, η^2^ = 0.12, respectively) (Fig. 4).

When using the regression model earlier described, we also found that the full-term group had a larger propSP compared to the very preterm group β_2_= −0.17 (p < 0.001). Additionally, the regression showed that both groups of infants had larger propSP values at 4 months than at 2 months CA β_1_ = 0.19 (p < 0.001). However, no interaction effect was found (β_3_) for propSP, i.e. the full-term group performed better than the very preterm at two as well as 4 months CA and both groups improve equally as they grow older. There was no difference in the share of very preterm infants reaching the 10th and 50th percentile of the full-term infants (respectively) at 2 and 4 months CA.

When investigating Gain with the regression analysis, the full-term group had a higher level of Gain compared to the very preterm group β_2_ = −0.25 (p < 0.001). Both groups of infants had higher Gain at 4 months than at 2 months CA β_1_ = 0.12 (p = 0.022). Even though no interaction effect was found (β_3_) for the whole group, a larger share of very preterm infants had reached the 10th (p < 0.001) and 50th (p = 0.030) percentile for the full-term infants at 4 months than at 2 months CA regarding Gain (Table 1).

**Table 1:** Change in gain of smooth pursuit between 2 and 4 months CA in the probability of very preterm infants reaching different percentiles of the full-term infant

	Share 2 months CA	Share 4 months CA	p-value for difference: 4–2 months
10th percentile	0.30	0.59	0.00
50th percentile	0.09	0.23	0.03

All estimates come from separate regressions (and include a constant), where the outcome variable is an indicator on whether very preterm infants reach p10/p50 in the full-term distribution. Standard errors are robust for heteroscedasticity and clustered on children to account for inter-child serial correlation.

**Figure 5:**
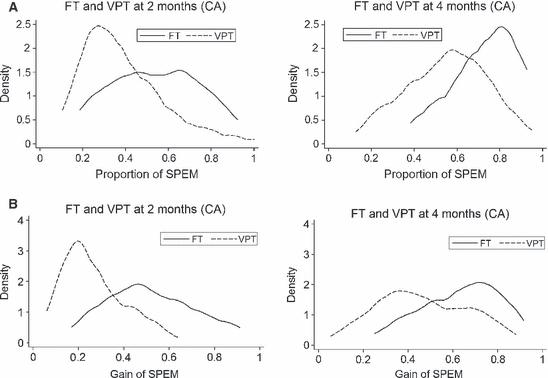
(A) Distribution density of proportion of smooth pursuit in full-term and very preterm infants at 2 and 4 months corrected age. (B) Distribution density of gain of smooth pursuit in full-term and very preterm infants at 2 and 4 months corrected age.

At 2 months CA, the very preterm infants had a more narrow distribution than the full-term group. As smooth pursuit developed, the distribution at 4 months CA for the full-term group tended to gather and approach adult levels whereas the distribution of the very preterm group became more dispersed (Fig. 5A, B).

**Figure 6:**
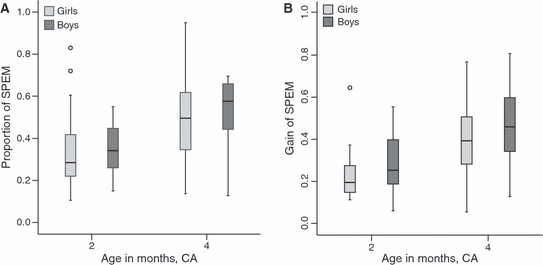
(A) Proportion of smooth pursuit in very preterm girls and boys at 2 and 4 months corrected age. (B) Gain of smooth pursuit eye movements of smooth pursuit in very preterm girls and boys at 2 and 4 months corrected age.

At 2 months CA, there was no significant difference between very preterm boys and girls regarding propSP, while the boys performed better in terms of Gain (p = 0.048). At 4 months CA, the very preterm boys showed both significantly larger propSP (p = 0.041) and higher Gain (p = 0.028) compared to the very preterm girls (Fig. 6A, B). No significant difference between boys and girls or between the tested sample and the whole LOVIS population in terms of gestational age, BW or neonatal complications were found.

This difference in gender was not found in the full-term group, i.e. there was no significant difference between girls and boys except for Gain at 4 months, where instead girls performed better than boys (p = 0.031).

## Discussion

In the present study, infants born very preterm (<32GW) showed lower Gain as well as propSP at 2 and 4 months CA compared to infants born at term. Both Gain and propSP are proportional measures where Gain measures smooth pursuit relative to the object motion and propSP measures smooth pursuit relative to the total eye movement. If the eye movements correspond to the object motion, then the two measures give the same results. If the head contributes substantially to the tracking, the Gain measure tends to underestimate the contribution of smooth pursuit in relation to the total eye movement. This is because head tracking is also smooth but it does not enter into the Gain equation. On the other hand, if the tracking is incomplete and the eyes fail to stabilize on the object, the propSP will overestimate the contribution of smooth pursuit in relation to the object motion. In this study, both measures are needed because very preterm and full-term infants may employ different amounts of head movements and eye movements in the attempt to stabilize gaze on the object. Although starting at a much lower level, as a group, the very preterm infants did not improve more than the full-term infants. Nevertheless, the number of very preterm infants who had reached the full-term infants’ level of Gain at 4 months CA had increased compared to 2 months CA while this was not the case for propSP. This result could be an effect on the fact that the very preterm group employed very small amount of raw eye movements at 2 months CA. In addition, the very preterm density curves became more dispersed at 4 months CA compared to 2 months CA. A plausible explanation for these findings is that some of the infants in the very preterm group caught up whereas some did not develop at all.

The results raise questions about the genesis of these impairments/delays. One possible cause could be disturbances in the development of motion perception which is crucial for smooth pursuit (11,24). It is possible that the origin of this disturbance could be found in the subcortical pathway, which is the dominating pathway for motion processing in 2 to 4-month-old infants (12). The very preterm infants employ less smooth pursuit on average, although some perform at full-term infants’ level. Whether these differences are caused by delayed development because of prematurity per se or by injuries incurred during the neonatal period remains to be investigated.

The impaired/delayed development of oculomotor capability described in this study has not been shown earlier. Previous studies report that preterm infants born 25.0–30.9 GW have more mature visual behaviour at 35 and 40 weeks postmenstrual age than infants born at 38–42 GW (25). This was measured as spontaneous ocular motility, ocular movements with target, attention at distance, ability to discriminate stripes, reaction to a coloured contrast target, tracking and fixation. It was suggested that preterm infants are exposed to visual stimuli at an earlier age than infants born at term and this might imply earlier development of visual function. However, the methods used in those studies to evaluate oculomotor ability could not make a distinction between different kinds of eye movements. Smooth pursuit is tied to attention and to the ability to predict upcoming motion and is an important component of eye movements in an environment that asks for capability to react on moving stimuli (10).

Lesions such as periventricular leukomalacia in the dorsal stream may cause impaired motion perception (26). This ability is anatomically related to structures important for recognizing faces and face expressions, and congenital prosopagnosia may accompany the deficit to see biological motion (27). These two functions are activated in the right temporal lobe, more close to the ventral stream, where early brain lesions may occur in preterm infants. Such damage impairs the ability to recognize faces, which normally develops at around 4 months of age (28) and continues to develop during the first year (29). In the present study, the object was a face-patterned stimulus. If some preterm infants had impaired ability for face detection, that might have decreased their attention to track the object.

Impaired or delayed development of smooth pursuit could imply that the infant cannot react and act functionally in daily situations where eye tracking is a prerequisite. This could affect the development of executive functions, coordination and perception, functions that preterm infants exhibit a greater risk of (4,5). Thus, it seems to be of interest to evaluate whether measures of smooth pursuit could be used to predict later impaired visuomotor capability. An early diagnosis could help the families and pedagogues in school to create adequate tools for adjustment of the children's milieu and evaluation of smooth pursuit might help to create a base for the development of more adequate direct interventions to support the infants.

The very preterm boys showed higher gain of smooth pursuit than the very preterm girls at both ages which is in contrast to several studies showing that preterm boys are affected with more neurodevelopment impairments than preterm girls (30). In this study group, there was no significant difference between girls and boys regarding gestational age, birth weight or incidence of SGA, BPD, ROP, IVH or PVL. Although the obtained gender difference is rather small, it is important to examine this difference in more detail in the future.

An advantage of the present study is that it is part of a long-term, population-based and prospective study on the same group of very preterm infants, including MRI and repeated ophthalmological, neuromotor and psychosocial examinations up to school age. This will enable us to study whether the disturbed development of smooth pursuit in early infancy is a predictor of future perceptual problems. In addition, all examinations were performed at one centre, in a standardized way, by experienced paediatricians, ophthalmologists, child radiologists, physiotherapists and staff at the Department of Psychology. The wide range in gestational ages, birth weights and neonatal complications are challenges for further investigations and analyses of risk factors affecting outcome in visuomotor capacity.

In conclusion, this report shows that visual oculo-motor development as measured by smooth pursuit is markedly delayed in very preterm infants at both 2 and 4 months CA. It is still unknown whether the gain and/or proportion of smooth pursuit in very preterm infants improve to the level of typically developing infants and if so, when this occurs. Whether the delay may predict later visual perceptual problems remains to be shown.

## Abbreviations

BPD: Bronchopulmonary dysplasia
CA: Corrected age
Gain: Gain of smooth pursuit eye movements
GW: Gestational week
IVH: Intraventricular haemorrhage
LOVIS: Longitudinal study of visuomotor capacity in very preterm-born infants
NEC: Necrotizing enterocolitis
propSP: Proportion of smooth pursuit eye movements
PVL: Periventricular leukomalacia
ROP: Retinopathy of prematurity
SGA: Small for gestational age
